# Suitability of Metal Block Augmentation for Large Uncontained Bone Defect in Revision Total Knee Arthroplasty (TKA)

**DOI:** 10.3390/jcm8030384

**Published:** 2019-03-19

**Authors:** Kwan Su Kang, Trinh Ngoc Tien, Myung Chul Lee, Kwon-Yong Lee, Bongju Kim, Dohyung Lim

**Affiliations:** 1 Department of Mechanical Engineering, Sejong University, Seoul 05006, Korea; oioi1578@kbiohealth.kr (K.S.K.); tientrinh.ute@gmail.com (T.N.T.); kwonlee@sejong.ac.kr (K.-Y.L.); 2 Department of Research & Development, Medical Device Development Center, OSONG Medical Innovation Foundation, Cheongju 28160, Korea; 3 Department of Orthopedic Surgery, College of medicine, Seoul National University, Seoul 03080, Korea; leemc@snu.ac.kr; 4 Dental Life Science Research Institute, Clinical Translational Research Center for Dental Science, Seoul National University, Seoul 03080, Korea; 5 Department of Research & Development, RNX Co., Ltd, Seoul 05006, Korea

**Keywords:** revision total knee arthroplasty, metal block-augmentation configuration, medium uncontained bone defect, large uncontained bone defect, finite element analysis

## Abstract

This study was performed to determine whether metal block augmentation is suitable for large uncontained bone defect via evaluations of differences in biomechanical characteristics among the configurations of metal block augmentations for medium or large uncontained bone defects in revision total knee arthroplasty (TKA). Three-dimensional finite element (FE) models of the proximal tibia with revision TKA were developed and analyzed considering the configurations of the metal block augmentations for medium and large uncontained bone defects. To identify differences in biomechanical characteristics according to the configurations of metal block augmentations, the stress transfer, strain distribution, and peak von Mises stresses (PVMSs) were assessed. Large and medium uncontained bone defects had similar ranges of strain below the critical bone-damage strain for the metal block augmentations, but the strain distribution characteristics differed in response to the metal block-augmentation configurations. PVMSs exceeding the yield strength of the bone cement for the single metal block-augmentation configurations were, on average, 1.4 times higher than those for double metal block-augmentation configurations for both medium and large uncontained bone defects. These findings suggest that metal block augmentation may be suitable for large uncontained bone defects (≤20 mm), compared with the results obtained for metal block augmentation used in medium uncontained bone defects (≤10 mm). If possible, double metal block augmentation is recommended for both medium and large uncontained bone defects rather than single metal block augmentation. It is also recommended that the metal block augmentation should be customized to meet the contact characteristics with the cortical bone, thereby ensuring better stress transfer and reducing the risk of the bone resorption due to stress shielding and bone-cement failure.

## 1. Introduction

With the development of new biomaterials, as well as surgical techniques, total knee arthroplasty (TKA) has become a favored method to enhance patient quality of life. However, failures of TKA have been reported [1]. Dalury et al. [2] and Sharkey et al. [3] reported that the most common reasons underlying TKA failure were loosening, infection, instability, periprosthetic fracture, and arthrofibrosis. Therefore, the number of revision TKAs is increasing [4,5]. Cram et al. [1] reported a 105.9% increase in the volume of revision TKAs, while the per capita utilization of revision TKA increased by only 56.8% between 1991 and 2010 among Medicare beneficiaries in the USA. Koh et al. [5] reported that the annual number of revision-TKA procedures increased by more than 23% from 2008 to 2012 in the Republic of Korea. Based on this tendency, it was reported that the annual economic burden of revision TKA will exceed $13 billion annually by 2030 compared to $2.7 billion in 2012 [1].

During revision TKA, reconstruction of uncontained bone defects is frequently required because the failure of TKA is often associated with loss of bone from either the proximal aspect of the tibia or the distal aspect of the femur [6,7]. Metal augmentation is one of the major options for the reconstruction of uncontained bone defects because of its extensive modularity, quick and easy use, minimal resection, and ready availability [8,9,10]. Metal block augmentation is currently one of the most common countermeasures for uncontained bone defects [10]. Baek et al. [11] used 4–5-mm single metal block augmentation in 29 cases, 8–10-mm single metal block augmentation in 35 cases, and double metal block augmentations in three cases. At the last follow-up, radiolucent lines were observed in four cases receiving 5-mm metal block augmentation, three cases receiving 8-mm metal block augmentations, and in no cases receiving double metal block augmentation. Patel et al. [12] reported radiolucent lines in 14% of 79 cases of revision TKA using metal block augmentation (4 or 8 mm). Recently, metal block augmentation with single or double metal blocks has been used for large uncontained bone defects (≤15 mm) [12,13], although it generally has been recommended only for small (≤5 mm) to medium (≤10 mm) uncontained bone defects [6,14]. However, there have been insufficient clinical follow-up and biomechanical studies comparing metal block augmentation for small to medium uncontained bone defects, resulting in ambiguity regarding the surgical effects on the use of metal block augmentation for large uncontained bone defects. The presence of a radiolucent line has also been suggested to occur with the use of metal block augmentation for large uncontained bone defects [15]. Chung et al. [16] reported radiolucent lines in three cases (17.6%) among 17 revision TKAs using double metal block augmentation (10 mm + 5 mm metal block augmentation and 10 mm + 10 mm metal block augmentation) for large uncontained bone defects. Therefore, reconstruction of large uncontained bone defects with metal block augmentation is a major challenge to improving the prognosis and longevity of revision TKA [6,7].

The main aims of the present study were to determine whether metal block augmentation was suitable for large uncontained bone defects by comparison with medium uncontained bone defects from a biomechanical view point, and to identify any differences in biomechanical characteristics among the configurations of metal block augmentation (single/double metal blocks) for medium or large uncontained bone defects.

## 2. Materials and Methods

### 2.1. Finite Element Models of the Proximal Tibia

#### 2.1.1. Finite Element Models of the Proximal Tibia

**Figure 1:**
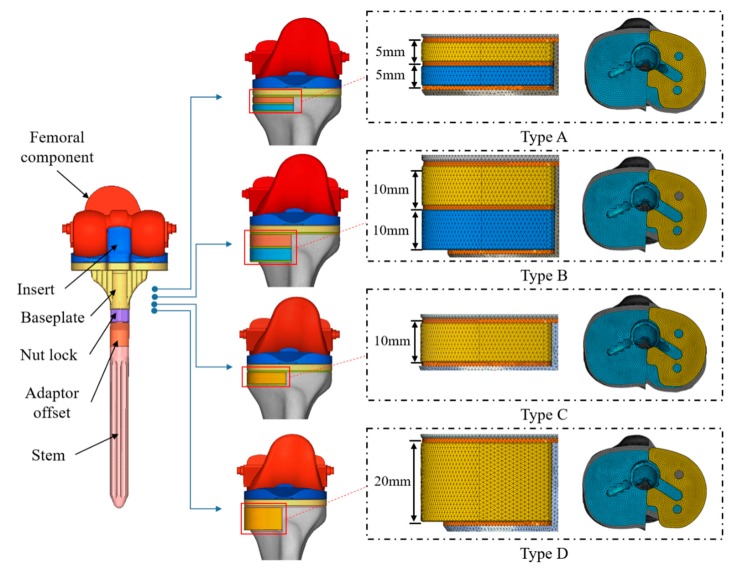
The configurations of the revision TKA with the metal block augmentations for the medium and large uncontained bone defects. Type A: Double metal block augmentation for medium uncontained bone defect, Type B: Double metal block augmentation for large uncontained bone defect, Type C: Single metal block augmentation for medium uncontained bone defect, Type D: Single metal block augmentation for large uncontained bone defect.

A three-dimensional (3D) computer-aided design (CAD) model of the tibia was developed based on a composite tibia model (#3401; Sawbones, Pacific Research Labs, Vashon, WA, USA). Resection of the tibia model was performed for revision TKA with metal block augmentation according to the traditional surgical procedure [17]. Here, resection of the tibia model was performed first at 8 mm below the medial articular surface of the tibia perpendicular to the mechanical axis of the tibia and second to achieve a 6° posterior slope of the baseplate of the tibia component for revision TKA surgery (Figure 1). Additional resections were performed to generate uncontained medium (10 mm depth) and large (20 mm depth) bone defects on the medial region of the tibia for placement of the metal block augmentations (Figure 1). Three finite element (FE) models of the tibia were developed based on the resected tibia models. Here, one FE model without uncontained bone defects was used to validate the FE analysis, while two FE models with uncontained bone defects were used to identify our study aims. The FE model used for validation was then developed using the four-node tetrahedral element (C3D4), which finally consisted of 1,067,544 elements and 192,766 nodes. Other FE models with uncontained bone defects were developed using the same element, which finally consisted of 1,089,060–1,140,598 elements and 220,610–229,198 nodes. The material properties of the tibia applied to the FE models are shown in Table 1 and were assumed to be linear elastic, isotropic, and homogenous.

#### 2.1.2. Finite Element Models for Revision TKA Components

Posterior-substituting (PS) fixed-bearing-type revision and metal block augmentation TKA (component size: #7; stem extension size: ⌀14, L120; adaptor offset: ⌀14, L60; Corentec Corp., Seoul, Republic of Korea) were used in the present study (Figure 1). Here, the PS fixed-bearing-type revision TKA was developed by adjusting factors such as the anterior flange roundness, femoral spherical condyle, bearing surface, and contour [18]. Specifically, the anterior flange roundness and femoral condyle shape were modified for high, deep flexion and sufficient posterior rollback, and the bearing surface was designed to reduce contact stress and achieve sufficient posterior rollback. The FE models for revision-TKA components and the metal block augmentations were developed using 3D CAD models. The FE models of the femoral and tibia components were then made up of 189,196 elements and 40,453 nodes and 100,688 elements and 22,284 nodes, respectively. The FE models of the baseplate and stem extension system of the tibia component were composed of 53,388 elements and 14,704 nodes and 86,573 elements and 20,025 nodes, respectively. Three FE models for the metal block augmentations were developed to fill the specific uncontained bone defects considered in the present study: Small metal block (5 mm thick), medium metal block (10 mm thick), and large metal block (20 mm thick). The FE models of the metal block augmentations were then composed of 21,885–67,839 elements and 5,594–14,896 nodes. Here, the FE models for revision TKA components and metal block augmentations were developed using the four-node tetrahedral element (C3D4). The material properties of the revision TKA components and the metal block augmentations applied to the FE models are shown in Table 1 [19] and were assumed to be linear elastic, isotropic, and homogenous.

#### 2.1.3. Insertion, Alignment, and Configuration of Revision TKA with Metal Block Augmentation

The insertion of the revision TKA components and the metal block augmentations into the tibia was performed following the traditional revision-TKA surgical guidelines, particularly the mechanical alignment strategy. The tibia baseplate component was placed in such a way that there was no protrusion over the cortical rim of the resected surface of the proximal tibia. The rotational alignment of the baseplate of the tibia component was then performed using the medial third of the tibia tuberosity landmark technique [20]. The long intramedullary stem with offset adaptor was aligned parallel to the stem of the tibia baseplate. The metal block augmentations were finally aligned with the conditions of the uncontained bone defects considered in the present study (Figure 1). Here, two small (5 mm + 5 mm thick, type A) and two medium (10 mm + 10 mm thick, type B) metal block augmentations were aligned for the medium and large uncontained bone defects, respectively, to achieve metal block augmentations with double metal blocks. One medium (10 mm thick, type C) and one large (20 mm thick, type D) metal block augmentation were also aligned for medium and large uncontained bone defects, respectively, to achieve metal block augmentation with a single metal block. Bone cement (Stryker Orthopaedics, Mahwah, NJ, USA) was then used to increase fixation between the prosthetic device and bone (bone–cement interface), metal block augmentation and tibia baseplate (cement–implant interface), and the first and second metal block in the case of metal block augmentations with double metal blocks (interlayer). The thickness of bone cement was then assumed to be 1 mm based on the clinical data and the results of previous studies [21]. 

#### 2.1.4. Loading, Boundary, and Contact Conditions

**Figure 2:**
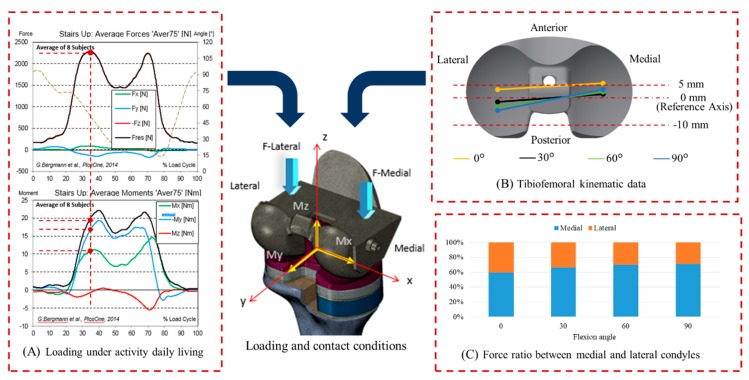
(**A**) Loading patterns for various activities of daily living (ADLs). The vertical force was selected with the maximum magnitude at desired flexion angle. The moment was selected corresponding to vertical force. (**B**) The contact locations between femoral and tibia insert components over the knee flexion angles. (**C**) The force ratio between the medial and lateral condyles for the knee flexion angles.

Six main physiological loading conditions (stand-up, sit-down, stair-up, stair-down, standing, and knee bend) generated frequently during activities of daily living (ADLs) were considered based on the public database reported by Orthoload (www.Orthoload.com) (Figure 2) [22]. The maximum joint reaction forces and their corresponding moments for the loading conditions considered were then selected and used for FE analyses. The loading conditions covered knee joint flexion angles of 0° to 90°. In addition, with the loading conditions, the tibiofemoral contact locations and the medial and lateral condyle force ratios were considered based on the results reported by Hanson et al. [23] and Sharmaet et al. [24] (Figure 2). The surface-to-surface contact algorithm was then applied to the contact surfaces of the FE models (e.g., femora–tibia component, bone cement–tibia component, bone cement–cancellous bone, bone cement–metal block augmentation, etc.) and the coefficients of friction applied to the contact surfaces were 0.01–1 as reported in previous studies (Table 2) [25,26,27]. A boundary condition was finally established to completely constrain six degrees of freedom of the distal end of the tibia. The FE models were finally solved using Abaqus 6.12 (Dassault Systèmes, Vélizy-Villacoublay, France).

### 2.2. Finite Element Model Validation

**Figure 3:**
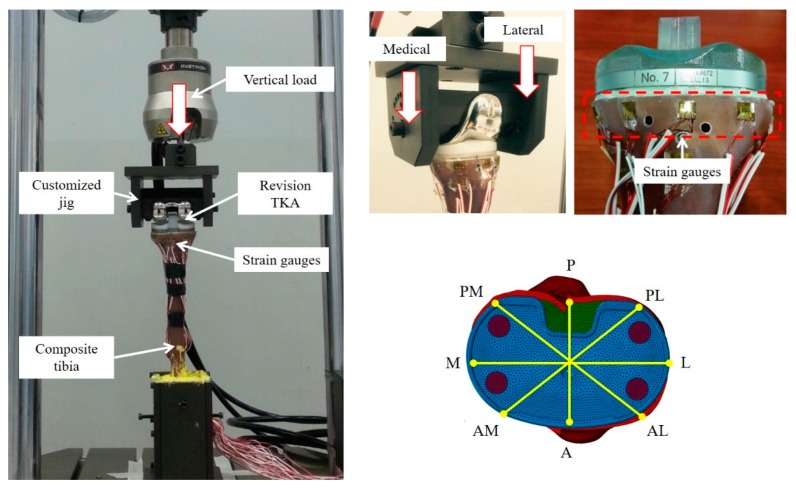
Mechanical test configuration for FE model validation. (A, anterior; M, medial; L, lateral; P, posterior; AM, anterior-medial; AL, anterior-lateral; PM, posterior-medial; PL, posterior-lateral region).

Actual mechanical tests based on the ASTM F1800 standard were performed to validate the FE model using a universal testing machine (Instron 8872; Instron, Norwood, MA, USA) (Figure 3). The composite tibiae with revision TKA without metal block augmentation were used for the actual mechanical tests (*n* = 6). Here, resection of the composite tibia for revision TKA, as well as insertion and alignment of the revision-TKA components, were performed following the same procedure used for FE model development. The composite tibiae with revision TKA and the femoral component were mounted onto the customized jig attached to the universal testing machine. The customized jig was designed specifically to represent knee flexion angle in the range of 0° to 140° considering the rollback phenomenon [28]. Eight half-bridge-type strain gauges (CAS Corp., Seoul, Republic of Korea) were attached to the surface of the proximal tibia near the resected surface of the tibia (Figure 3). A vertical load of 2100N (3 × 70 kg body weight (BW)) through the femoral component was finally applied to the composite tibia with revision TKA. A medial and lateral condyle force ratio (6:4) was considered based on a previous study [29]. FE analysis was then performed for validation under the same loading and boundary conditions that were used in the actual mechanical tests with Abaqus 6.12. The FE model was validated by comparing the strains obtained from the strain gauges with those determined by FE analysis. Here, eight regions of interest (ROIs), which were located in the same anatomical areas (i.e., locations of strain gauge attachment) considered in the actual mechanical tests, were used to compare the strains obtained from the mechanical tests with those from FE analysis. 

### 2.3. Data Analyses

**Figure 4:**
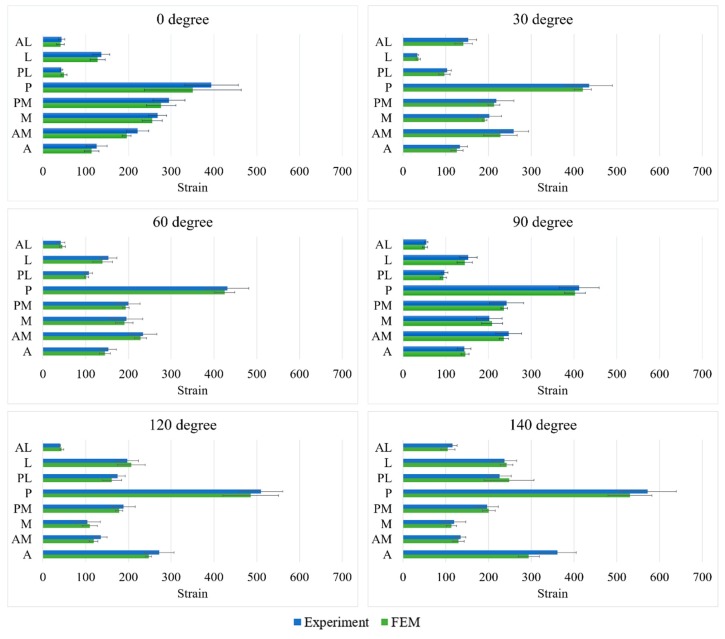
Results of the FE model validation (comparison of the strains from the FE models with those obtained from mechanical tests). (A, anterior; M, medial; L, lateral; P, posterior; AM, anterior-medial; AL, anterior-lateral; PM, posterior-medial; PL, posterior-lateral regions).

The principal stress flow inside the tibia was analyzed to characterize stress transfer in the tibia during motions associated with ADLs based on the metal block-augmentation configurations (types A–D). The distribution of strain on the resected surface of the cortical bone of the proximal tibia immediately below the metal block augmentation was characterized to predict the possibility of tibia bone resorption. The possibility of bone resorption related to radiolucent line was then analyzed by comparing the critical bone-damage strain (≤200 µstrain), which is capable of reducing the capacity for bone remodeling, leading to bone degeneration based on Frost’s mechanostat theory [30]. Here, five ROIs on the resected surface were considered to quantitatively determine the strain distribution characteristics (Figure 4). Finally, the possibility of osteolysis in the tibia was analyzed by predicting the possibility of bone-cement failure. Here, the possibility of bone-cement failure was analyzed by comparing peak von Mises stress (PVMS) inside the bone cement calculated by FE analysis to the yield strength (21MPa) of bone cement.

## 3. Results

### 3.1. Finite Element Model Accuracy

The strains computed from FE analyses and measured in mechanical tests are shown in Figure 4. Close correspondences between strains computed from FE analyses and those measured in mechanical tests were observed at the medial, posterior-medial, and posterior regions of the proximal tibia, with mean differences not exceeding 7%. However, the differences were relatively high at slightly anterior (8.3% ± 5.9%), anterior-medial (7.8% ± 4.7%), anterior-lateral (7.7% ± 2.2%), and posterior-lateral (7.6% ± 3.9%) regions. 

### 3.2. Principal Stress Flow within Cortical Bone of the Tibia

**Figure 5:**
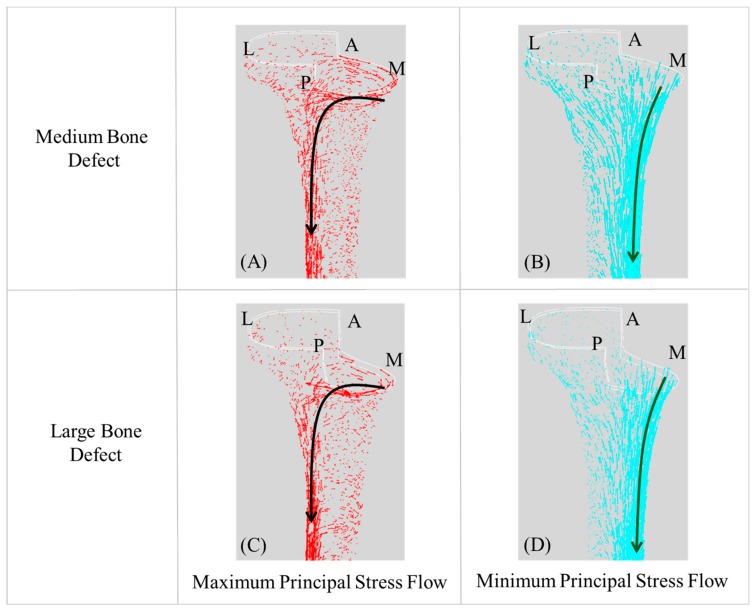
Representative maximum principal stress flows for the metal block augmentations of the (**A**) medium and (**C**) large uncontained bone defects and minimum principal stress flows for the metal block augmentations of the (**B**) medium and (**D**) large uncontained bone defects anterior cortex line. (A, anterior; M, medial, L, lateral; P, posterior regions).

The maximum and minimum principal stress flows within the cortical bone of the tibia are shown for revision TKAs with metal block augmentations applied for medium and large uncontained bone defects (types A–D) (Figure 5). The directions of maximum and minimum principal stress general flows had similar patterns among types A–D. However, the maximum and minimum stresses in types A and C were approximately double those in types B and D. The maximum principal stresses in types A and C were generally higher than those in types B and D in two regions beneath the resected surface of the medial side of the cortical bone and on the interosseous border of the tibia. The minimum principal stresses in types A and C were higher than those in types B and D within the medial border of the tibia. 

### 3.3. Strain Distribution on Cortical Bone of Proximal Tibia below Baseplate

**Figure 6:**
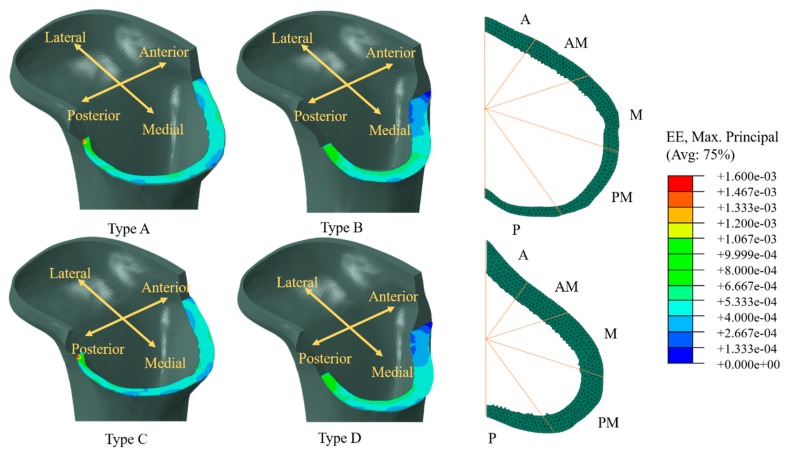
Representative strain distributions on the resected surface of the cortical bone of the proximal tibia for the metal block augmentations of the medium and large uncontained bone defects. Type A: Double metal block augmentation for medium uncontained bone defect, Type B: Double metal block augmentation for large uncontained bone defect, Type C: Single metal block augmentation for medium uncontained bone defect, Type D: Single metal block augmentation for large uncontained bone defect. (A, anterior; M, medial; L, lateral; P, posterior regions).

The strain distributions on the resected surface of the cortical bone of the proximal tibia are shown for revision TKAs with metal block augmentations for medium and large uncontained bone defects (types A–D) (Figure 6). The maximum strains generally occurred at the posterior region of the resected surface of the cortical bone of the proximal tibia for types A–D, although the strain distribution characteristics were slightly different among revision TKAs with metal block augmentations for medium uncontained bone defects (types A and C) and for large uncontained bone defects (types B and D). In types A and C, the strains in the medial region were generally lower than those in the other regions, and the distribution of strains within the medial region that were below the critical bone-damage strain (200 μstrain) was 1.1–3.0 times higher than in the other regions. Here, there were generally small differences (<2%) in distributions of strains below the critical bone-damage strain between types A and C. In types B and D, the strains in the anterior region were generally lower than those in the other regions, and the distribution of strains within the anterior region of the resected surface of cortical bone of the proximal tibia that were below the critical bone-damage strain was 1.1–9.0 times higher than in the other regions. Small differences (<2%) in distributions of strains below the critical bone-damage strain were generally observed between types B and D. The range of strains below the critical bone-damage strain in metal block augmentations for large uncontained bone defects (types B and D) was similar to that for medium uncontained bone defects (types A and C).

### 3.4. Peak von Mises Stress within Bone Cement

**Figure 7:**
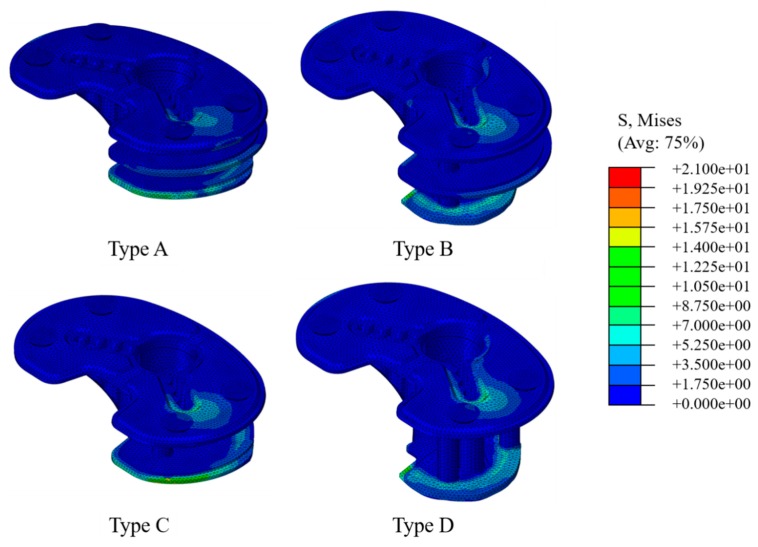
Representative stress distributions within the bone cements for the metal block augmentations of the medium and large uncontained bone defects. The red rectangle indicates the region identified by PVMS. Type A: Double metal block augmentation for medium uncontained bone defect, Type B: Double metal block augmentation for large uncontained bone defect, Type C: Single metal block augmentation for medium uncontained bone defect, Type D: Single metal block augmentation for large uncontained bone defect. (A, anterior; M, medial, L, lateral; P, posterior regions).

The distributions of von Mises stresses within the bone cements for types A–D are shown in Figure 7 and PVMSs are summarized in Table 3. The PVMSs for types A and C and types B and D were generally observed at the posterior-medial and the posterior regions of the last layer of bone cement, respectively. Under high physiological loading conditions, PVMSs ranged from 7.9 to 15.3 MPa for types A and C; PVMSs were 1.3–1.7 times higher in type C than in type A. Types B and D showed PVMSs of 8.9–17.5 MPa, and the PVMSs were 1.2 times higher in type D than in type B. PVMSs exceeding the yield strength of the bone cement for the single metal block-augmentation configurations (types C and D) were an average of 1.4 times higher than those for double metal block-augmentation configurations (types A and B) for both medium and large uncontained bone defects. Under low physiological loading conditions, PVMSs in types A and D did not exceed 7.3 MPa (approximately 1/3 the yield strength of bone cement). 

## 4. Discussion and Conclusions

The FE model used in this study showed a high degree of accuracy (93.8% ± 2.1%), although it had somewhat lower accuracy (85.8%–90.1%) in the anterior, anterior-medial, anterior-lateral, and posterior-lateral regions of the proximal tibia. This low accuracy was likely caused by differences between the material properties input into the FE model of the elastic element and the composite material actually used in the mechanical test and/or to erroneous attachment of strain sensors due to the morphological specificity of the posterior region of the proximal tibia. However, despite the differences between the results of FE analysis and actual mechanical tests, the accuracy of >85% for the FE models developed in this study suggested their high validity.

The flow of maximum and minimum principal stresses of the tibia was generally similar in the proximal and distal regions for revision TKAs with metal block augmentations for both medium and large uncontained bone defects (types A–D). In four different metal block augmentations used for revision TKA, the maximum principal stress flowed from the proximal medial to the distal lateral direction, and the minimum principal stress flowed from the proximal medial to the lateral direction. This was considered characteristic of the tibia bending moment caused by the load applied to the medial and lateral condyle of the tibia. However, there were differences in magnitudes of maximum and minimum principal stresses according to the size of the bone defect. These differences were thought to be caused by the effect of stress shielding due to the differences in thickness of metal block augmentations. The metal block augmentation acted as a barrier, impeding the flow of principal stress of the proximal tibia; when the bone defect was large, both the thickness of the metal block augmentation and the effect of stress shielding increased. These results were consistent with those reported previously by Martinet et al. [31] and Deenet et al. [32]. The results indicated that larger bone defects were associated with weaker bending moment of the tibia from lateral to medial due to stress shielding caused by the metal block augmentation. These changes in tibial proximal and distal principal stress can affect the ratio of deformation of the tibia and pubic bone injury (≤50 μstrain). 

The range of strains below the critical bone-damage strain in metal block augmentations for large uncontained bone defects (types B and D) was similar to that for medium uncontained bone defects (types A and C), but the strain distribution characteristics were different in relation to the metal block-augmentation configurations. These differences in strain distribution may have been due to the characteristics of contact between the metal block augmentation and the resected surface of the cortical bone of the proximal tibia. The metal block augmentations for medium and large uncontained bone defects partially and completely covered the cortical bone of the proximal tibia, respectively. However, the contact characteristics did not cause significant differences in the range of strain below the critical bone-damage strain under the physiological loads considered in the present study. In conclusion, although diverse biological and mechanical factors influence bone resorption [33], metal block augmentations may be suitable for the large uncontained bone defects ≤20 mm considered in the present study based on the results obtained with metal block augmentations for medium uncontained bone defects, from the viewpoint of bone resorption. However, as the strain distribution is influenced by the direction and magnitude of physiological load on the tibia, the ability to predict bone resorption based on the distribution characteristics for strains below the critical bone-damage strain is limited. For example, if the magnitude of the physiological load increases (e.g., the intensity of ADLs increases), the distribution characteristics for strains below the critical bone-damage strain will change independent of the metal block-augmentation configuration, thus reducing the likelihood of bone resorption. However, our results are meaningful at least for determining the relevance of the use of metal block augmentation for large uncontained bone defects via prediction of bone resorption based on the distribution characteristics of strains below the critical bone-damage strain.

The PVMS results within the bone cement suggested the likelihood of bone-cement failure. PVMSs exceeding the yield strength of the bone cement for single metal block-augmentation configurations (types C and D) were, on average, 1.4 times higher than those for double metal block-augmentation configurations (types A and B) for both medium and large uncontained bone defects. These observations suggested that the stress flow characteristics responded to differences in configuration (single or double) of the metal block augmentation. That is, the stresses generally flowed from the metal block to the bone cement directly in the case of single metal block augmentation, unlike double metal block augmentation. PVMSs exceeding the yield strength of the bone cement were generally detected in the posterior region of the last layer of the bone cement for large metal block augmentation and in the posterior-medial region of the last layer of bone cement for medium metal block augmentation. These observations were likely due to the characteristics of contact between the metal block augmentation and the cortical bone. Large metal block augmentation showed incomplete coverage in the posterior region of the resected surface of the proximal tibia. For medium metal block augmentation, there was an area of incomplete coverage in the posterior-medial region of the resected surface of the proximal region. In the case of a large bone defect, there was an area of incomplete coverage in the posterior region. These contact characteristics may have been important factors in increasing stress. These findings suggest that the number of metal blocks for metal augmentation configuration and the contact characteristics between the metal block and the cortical bone are important biomechanical factors related to bone-cement failure. Bone-cement failure generates bone cement debris, thus resulting in inflammation and osteolysis (bone resorption) in the tibia [33]. In conclusion, the results presented above suggested that use of double metal block augmentations, rather than single metal block augmentations, for bone defects of the same size may reduce the risk of the bone-cement failure, consequently decreasing the risk of tibia inflammation and osteolysis. In addition, use of a customized metal block that can completely cover the cortical bone area in contact with the bone cement may be recommended to reduce the risk of bone-cement failure. However, as PVMS within the bone cement is influenced by the direction and magnitude of physiological loads on the tibia, the ability to accurately predict the likelihood of bone-cement failure or osteolysis is limited. For example, if the magnitude of physiological load increases (e.g., increase in intensity of ADLs), the PVMS will increase regardless of the metal block-augmentation configuration, thus increasing the likelihood of bone-cement failure and osteolysis. However, our results were meaningful for identification of the biomechanical factors that can influence bone-cement failure with the use of metal block augmentations.

In conclusion, metal block augmentations may be suitable for large uncontained bone defects (10–20 mm) based on the results obtained with metal block augmentation for medium uncontained bone defects (≤10 mm), and the double metal block-augmentation configuration is recommended for both medium and large uncontained bone defects rather than single metal block augmentation, if possible. Metal block augmentation should be customized to achieve full contact with the cortical bone to ensure better stress transfer and thus reduce the risk of bone resorption by stress shielding and bone-cement failure. 

This study had several limitations. We did not consider the clinical factors affecting the results of the present study, including incomplete analysis of the likelihood of bone resorption (osteolysis) based on a single value (criterion) despite the influence of diverse biological and mechanical factors; nor did we evaluate clinical efficacy due to difficulty in performing longitudinal follow-up evaluations of patients within a short period. Further studies are required to address these limitations. Nevertheless, the results of the present study are useful for identifying the biomechanical characteristics associated with successful outcomes of revision TKA with metal block augmentation for medium and large uncontained bone defects.

## Figures and Tables

**Table 1:** Material properties for the cortical and cancellous bone and revision TKA components.

Part of FE Model	Material	Elastic Modulus (MPa)	Poisson’s Ratio
Cortical	Cortical bone	17,000	0.36
Cancellous	Cancellous bone	300	0.3
Baseplate	Cobalt-chromium alloy (CoCr)	200,000	0.33
Femoral			
Nut adaptor			
Adaptor offset	Titanium alloy	113,000	0.33
Extension stem			
Block augment			
Spacer	Ultra-high molecular wear polyethylene (UHMWPE)	900	0.46
Bone cement	Polymethylmethacrylate (PMMA)	2280	0.3

**Table 2:** A coefficient of the friction applied to the surface contact conditions.

Interaction	Coefficient of Friction
Femoral-tibial insert contact	0.01
Cement-tibial baseplate	0.4
Cement-metal block augmentation	0.25
Cement-bone	1
Extension stem-bone	0.25

**Table 3:** PVMS within bone cement.

Type of Loading	ADL (Knee Flexion Angle)	Cement Layer	PVMS (MPa)			
			Type A	Type B	Type C	Type D
Low	Knee bend (0°)	First	2.6	3.3	3.4	3.3
		Second	1.7	1.3	-	-
		Third	4.4	7.3	5.4	7.2
	Stand up (0°)	First	2.6	3.2	3.3	3.3
		Second	1.6	1.3	-	-
		Third	4.3	7.1	5.3	7.0
	Sit down (30°)	First	3.4	4.1	4.2	4.4
		Second	2.2	1.6	-	-
		Third	4.3	5.2	6.2	6.0
	Stair up (30°)	First	1.2	1.5	1.6	1.6
		Second	0.8	0.6	-	-
		Third	2.0	2.0	2.3	2.4
High	Standing (0°)	First	5.9	7.6	7.4	7.8
		Second	3.3	2.1	-	-
		Third	11.9	14.9	13.2	17.5
	Stair down (30°)	First	6.9	8.6	8.6	9.1
		Second	3.9	2.6	-	-
		Third	9.7	10.8	14.2	12.2
	Stair up (60°)	First	7.3	10.4	9.3	11.3
		Second	4.3	2.8	-	-
		Third	10.4	13.4	15.3	12.1
	Sit down (90°)	First	6.9	7.6	7.4	8.9
		Second	3.3	2.2	-	-
		Third	4.9	8.9	13.5	10.1
	Stand up (90°)	First	7.7	9.7	8.4	9.2
		Second	3.9	3.4	-	-
		Third	8.9	9.8	14.9	11.2
